# Horseshoe perianal abscess with deep gluteal extension managed by multi-incision and ultrasound-guided catheter drainage: a case report

**DOI:** 10.1093/jscr/rjaf885

**Published:** 2026-03-21

**Authors:** Ruirui Liu, Jiaqin Li, Yi Zhao, Shenglan Zhong, Runyi Geng, Hao Xu

**Affiliations:** Anorectal Department, Shanghai Fourth People’s Hospital Affiliated to Tongji University, No. 1279 Sanmen Road, Hongkou District, Shanghai 200434, China; Anorectal Department, Shanghai Fourth People’s Hospital Affiliated to Tongji University, No. 1279 Sanmen Road, Hongkou District, Shanghai 200434, China; Anorectal Department, Shanghai Fourth People’s Hospital Affiliated to Tongji University, No. 1279 Sanmen Road, Hongkou District, Shanghai 200434, China; Anorectal Department, Shanghai Fourth People’s Hospital Affiliated to Tongji University, No. 1279 Sanmen Road, Hongkou District, Shanghai 200434, China; Anorectal Department, Shanghai Fourth People’s Hospital Affiliated to Tongji University, No. 1279 Sanmen Road, Hongkou District, Shanghai 200434, China; Anorectal Department, Shanghai Fourth People’s Hospital Affiliated to Tongji University, No. 1279 Sanmen Road, Hongkou District, Shanghai 200434, China

**Keywords:** perianal abscess, horseshoe abscess, deep gluteal abscess, ultrasound-guided drainage, anal gland infection

## Abstract

Perianal abscesses are common anorectal infections typically confined to superficial spaces, but deep extension into adjacent compartments is rare and often underdiagnosed due to subtle early symptoms. We report a 68-year-old man with a complex horseshoe perianal abscess that extended into the deep gluteus maximus. Initial drainage targeted the anorectal spaces based on clinical and magnetic resonance imaging findings, yet postoperative fever and persistent pain prompted repeat imaging, which revealed a deep gluteal abscess. Ultrasound-guided catheter drainage was performed successfully without complications. This case illustrates an unusual pathway of infection spread from the anal gland through the intersphincteric space and ischiorectal fossae into the gluteal compartment. Contributing factors included delayed presentation, malnutrition, and immunosuppression. Comprehensive assessment, multidisciplinary management, and image-guided intervention were essential for a successful outcome. Clinicians should suspect deep gluteal extension in patients with persistent systemic signs despite perianal drainage to avoid diagnostic delay and improve outcomes.

## Introduction

Perianal abscesses are common anorectal infections, usually originating from cryptoglandular obstruction and most often confined to superficial spaces with localized symptoms [1]. Deep abscesses, such as those in the ischiorectal or supralevator compartments, are less frequent, but when present they may progress insidiously and rapidly cause systemic sepsis, organ dysfunction, or even death [2]. Extension into the gluteal muscles is exceptionally rare, with only isolated case reports describing this pathway of spread, often misdiagnosed or detected late owing to atypical clinical features.

In this case report, we present a 68-year-old man with a complex horseshoe perianal abscess that extended secondarily into the deep gluteus maximus, highlighting the diagnostic challenges and the role of multidisciplinary and image-guided management.

## Case report

**Figure 1:**
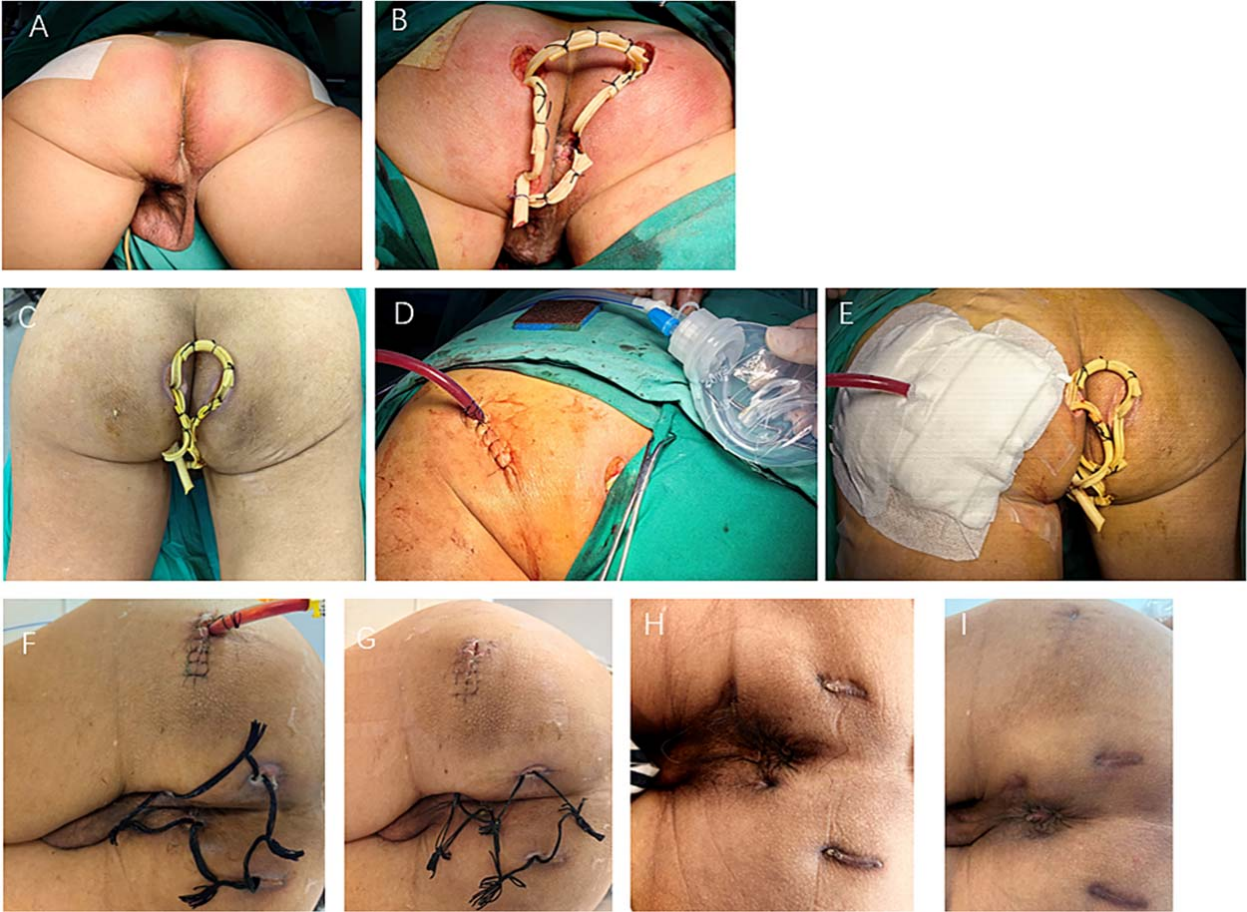
Clinical photographs from preoperative to follow-up stages. (A) Pre-op day 1: bilateral perianal swelling with erythema, induration, raised tense skin, and significant tenderness, without obvious fluctuation or skin breakdown. (B) Operative day: perianal abscess drainage. (C) Postoperative day 7: skin swelling and significant tenderness in the left gluteal and groin regions. (D) Postoperative day 7: negative pressure catheter drainage was performed to evacuate the deep gluteal abscess under spinal anesthesia. (E) Postoperative day 7: the deep gluteal abscess drainage. (F) Postoperative day 2: perianal drainage catheters were converted to lose seton. (G) Postoperative day 3: the gluteal catheter was successfully removed. (H) Postoperative day 8: complete recovery was achieved. (I) Postoperative day 6: no recurrence.

**Figure 2:**
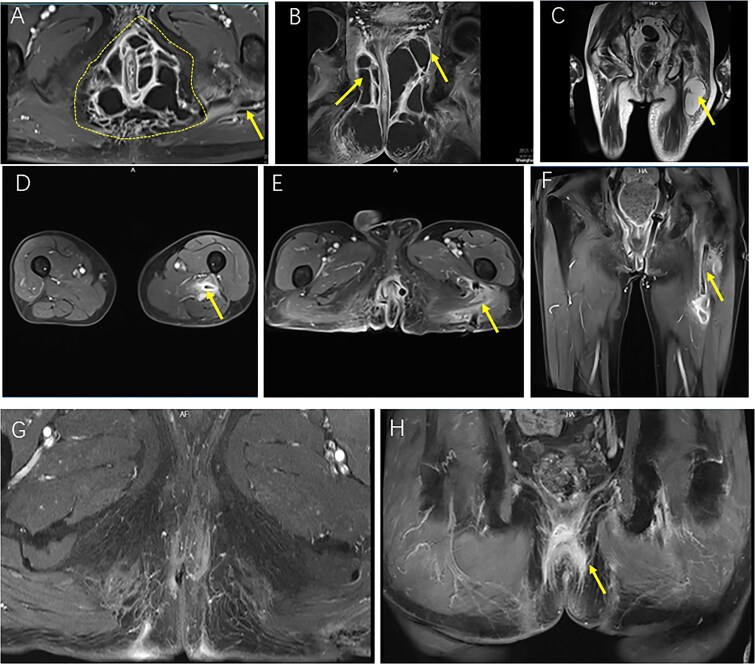
Sequential MRI images illustrating the preoperative extent, postoperative course, and resolution of a complex horseshoe perianal abscess with deep gluteal involvement following surgical drainage. (A, B) Preoperative CE FS-T1-TSE axial (A) and coronal (B) MRI images demonstrate a complex horseshoe-shaped perianal abscess involving both ischiorectal fossae (outlined by a dashed line). The abscess extends superiorly to the apex of the left ischiorectal fossa with associated inflammatory changes involving the left levator ani muscle (arrow). A small secondary gluteal abscess is also noted (arrow). (C) T2WI-FS coronal MRI five days postoperatively demonstrates a deep gluteal abscess (arrow). (D–F) Two-week postoperative CE FS-T1-TSE MRI shows the gluteal region in axial view (D), the perianal region in axial view (E), and the perianal region in coronal view (F), with the left ischiorectal fossa drain accurately positioned at the apex and marked reduction of inflammatory infiltration and abscess cavities. (G, H) Follow-up CE FS-T1-TSE MRI images obtained three months postoperatively in axial (G) and coronal (H) planes show near-complete resolution of inflammation. No residual abscess is observed (arrow).

A 68-year-old man without significant comorbidities such as diabetes or autoimmune diseases and without a history of long-term steroid use presented with a 2-week history of perianal swelling and fever. The condition acutely worsened over the past 4 days with increasing pain, malaise, and worsening local signs. Digital rectal and perianal examination revealed bilateral perianal swelling (~8 × 10 cm) with erythema, induration, raised tense skin, and significant tenderness, without obvious fluctuation or skin breakdown (Fig. 1A). Magnetic resonance imaging (MRI) revealed a complex horseshoe perianal abscess involving multiple anatomical spaces, including the perianal, ischiorectal, deep postanal, and intersphincteric spaces (Fig. 2A and B). Laboratory markers showed leukocytosis (WBC 30.15 × 10^9^/L), neutrophilia (86.4%), and markedly elevated CRP (191.65 mg/L), suggestive of severe infection. The patient experienced a decrease in appetite over the past two weeks due to perianal discomfort, which led to weight loss and signs of malnutrition, potentially contributing to an immunocompromised state.

### Surgical management and postoperative care

The patient underwent drainage of the perianal abscess under spinal anesthesia. Intraoperative exploration demonstrated extensive suppuration involving multiple anatomical spaces, with the deepest cavity located at the apex of the left ischiorectal fossa and extension to the levator ani muscle complex. Multiple small counter-incisions were created to achieve thorough debridement and dependent drainage, and Penrose drains were inserted through these tracts. A mushroom catheter was placed at the left ischiorectal apex, and ~220 ml of purulent fluid was evacuated (Fig. 1B).

Postoperatively, the patient developed a 3-day fever with left gluteal and posterior thigh discomfort. In view of ongoing sepsis, microbiological cultures were obtained from the abscess, and the results identified *Escherichia coli*. Based on antibiotic susceptibility testing, the pathogen was found to be sensitive to piperacillin-tazobactam, cefoperazone-sulbactam, ceftazidime, cefepime, and imipenem. As a result, antibiotic therapy was escalated to piperacillin-tazobactam and supportive measures included albumin replacement, electrolyte correction, and nutritional supplementation.

By postoperative day 5, although the fever had subsided, the patient reported worsening gluteal and thigh pain with impaired mobility and antalgic gait. Ultrasound and pelvic MRI confirmed a deep abscess within the gluteus maximus (Fig. 2C). Multidisciplinary consultation was obtained, and on postoperative day 7, ultrasound-guided catheter drainage under spinal anesthesia evacuated ~200 ml of purulent fluid without complications (Fig. 1C–E).

Follow-up MRI at 2 weeks demonstrated marked resolution, and perianal drains were converted to lose setons (Figs 1F and 2D–F). By postoperative week 3, the gluteal catheter was removed (Fig. 1G). Complete recovery was achieved by 8 weeks (Fig. 1H), and at 3-month MRI and 6-month clinical follow-up, no recurrence was detected (Figs 1I and 2G and H).

**Figure 3:**
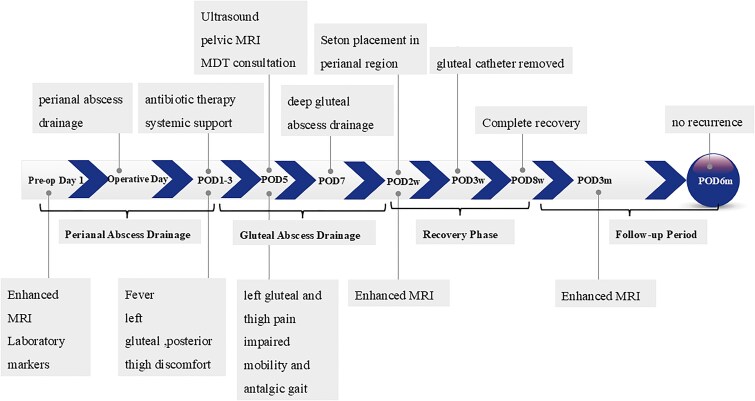
Clinical course from admission to recovery, showing major diagnostic and therapeutic milestones.

The overall sequence of clinical events, including both drainage procedures and follow-up assessments, is illustrated in Fig. 3.

## Discussion

Perianal abscesses most often arise from cryptoglandular infection and are typically confined to superficial anorectal spaces. Horseshoe variants represent a more complex form, spreading circumferentially through the intersphincteric space into bilateral ischiorectal fossae. Secondary extension into the gluteal region is exceptionally rare and only sporadically reported. Deep gluteal abscesses are usually linked to trauma, intramuscular injections, or skin infection; in this case, however, the origin was a cryptoglandular abscess that tracked along defined anatomical planes into the gluteus maximus [3].

The pathophysiology of spread warrants attention. Infection likely began in the anal gland, penetrated the internal sphincter, and formed an intersphincteric abscess [4, 5]. It then extended circumferentially, evolving into a horseshoe abscess, before transgressing the external sphincter into the ischiorectal fossae. Persistent infection and inflammatory pressure may have compromised fascial barriers, allowing dissection into the deep gluteal compartment and formation of a secondary abscess within the gluteus maximus.

This case highlights several important clinical lessons. First, early and thorough drainage is essential, as the deep gluteal abscess was initially overlooked due to nonspecific symptoms, underscoring the need for comprehensive preoperative evaluation [6]. Second, vigilant systemic management is critical—postoperative fever indicated persistent infection, requiring timely antibiotic escalation, correction of hypoalbuminemia, electrolyte imbalances, and nutritional support [7]. Third, multidisciplinary collaboration was indispensable for addressing the complex challenges of sepsis, metabolic disturbances, and malnutrition. Finally, ultrasound-guided drainage offered a safe, precise, and minimally invasive approach, minimizing iatrogenic risks in managing deep abscesses. These insights may guide clinicians in optimizing diagnosis and treatment of complex perianal infections.

In summary, this case demonstrates that perianal abscesses, particularly complex horseshoe variants, can extend into deep compartments such as the gluteus maximus, even in the absence of localizing symptoms. Clinicians should maintain a high index of suspicion for deep-seated infection when systemic signs or unexplained pain persist after standard drainage. Early recognition and timely intervention are essential for favorable outcomes. Key elements include prompt diagnosis, thorough surgical debridement, broad-spectrum antimicrobial therapy, repeated clinical reassessment, and adequate nutritional support. Multidisciplinary management and the use of ultrasound-guided drainage further enhance outcomes in these rare but clinically significant presentations.
